# Editorial: History of Growth Hormone: Animal to Human

**DOI:** 10.3389/fendo.2021.793272

**Published:** 2021-11-03

**Authors:** Edward O. Reiter, Laurie E. Cohen, Alan D. Rogol

**Affiliations:** ^1^ Department of Pediatrics, Baystate Medical Center, Springfield, MA, United States; ^2^ Boston Children’s Hospital, Harvard Medical School, Boston, MA, United States; ^3^ Pediatrics/Endocrinology, University of Virginia, Charlottesville, VA, United States

**Keywords:** growth, growth hormone, species specificity, childhood cancer, long-acting growth hormone, growth prediction, IGF-1

## Introduction

Fascination with extremes in the size of man or animals (as well, amazingly, of plants) has a long history stretching to Antiquity. Giants are described in the Bible (Goliath and the Nephilim), as well as portrayed in classical art, such as The Colossus of Goya (1808-1812), displayed in the Prado Museum. At the other extreme, the same museum portrays little people, Las Meninas by Velazquez. There is the well-known American, General Tom Thumb, who likely had GH deficiency (GHD) and was a successful member of the PT Barnum Circus.

The striking clinical picture of *acromegaly* with the suggestion of a pituitary mass led to studies in the 19^th^ and 20th centuries and the realization that a pituitary substance was responsible. Extraction and purification of a growth-promoting factor within the anterior pituitary was enabled by the development of successful hypophysectomy in animals. That was followed by the ability to purify various pituitary factors and administer them to reverse the post-hypophysectomy biological state. Additionally, it was important to develop sensitive bioassays. Using a cartilage bioassay, Knobil and colleagues demonstrated species specificity of primate and sub-primate GH (1).

## Human Growth Hormone Treatment

After hGH was purified, administration to an adolescent was initiated in 1956 by Raben and his associates (2). By the mid-1960’s, many children with GHD were being treated with hGH supplied by the NIH-funded National Pituitary Agency which coordinated the extraction and purification of hGH from cadaveric pituitaries (3). As the number of children with GHD increased and other indications for treatment of short children developed, the need for more hGH was apparent. At the same time, however, there was the realization that human pituitary glands were contaminated with the agent responsible for the nearly uniformly fatal neurodegenerative condition, Creutzfeldt-Jacob Disease (CJD). This quickly led to cessation of the production and administration of pituitary hGH. Fortuitously, we were at the dawn of the recombinant DNA era when an essentially unlimited supply of highly pure biosynthetic rhGH became available.

## Growth Hormone Deficiency

Detailed information regarding the diagnosis, treatment, and long-term outcomes is provided in the second manuscript in this Research Topic by Professor Ranke. Diminished GH production is primarily due to developmental abnormalities of the hypothalamic-pituitary area. Characterizing the action of GH has led to extensive examination of the GH-dependent peptides, IGF-I and IGFBP3, but beyond the scope of this presentation.

The incidence of diagnosed GHD has increased in parallel to the almost limitless availability of rhGH. That has enabled treatment regimens based on clinical need rather than to a constrained supply of pituitary-extracted hGH. Ranke describes the range of findings and the laboratory studies required to confirm the suspicion of impaired GH production. Decreased height velocity of children in infancy and early childhood may be difficult to ascertain, so the occurrence of hypoglycemia associated with other pituitary hormones must be included in the assessment. IGFBP3 measurements, which are less influenced by nutrition or illness than of IGF-I, may be used. Imaging studies of the hypothalamic-pituitary anatomy are particularly valuable in the very young child. During childhood, careful determination of diminished height velocity with ample interval between measurements is a central tenet. In addition to the auxologic findings, many different pharmacologic “provocative” tests evaluating GH secretion have been used to confirm GHD. As the child’s age approaches pubertal onset, but remains prepubertal, the impact of relatively low levels of sex steroids may impair GH secretion and yield results falsely low stimulated GH results suggesting impaired GH secretion. Auxologic data remain important at this stage but must be considered in light of pubertal status similar to the pharmacologic test results.

## Treatment of Children With GHD

Daily subcutaneous doses of rhGH at ranges of 25-43 µg/kg/day are used, bearing in mind adherence to this regimen to achieve optimal growth. Determination of the response to rhGH treatment is largely based on height velocity during treatment comparing growth data from large pharmaco-epidemiologic studies. Varied auxologic parameters have been inserted into sophisticated GH-treatment-associated prediction models that permit guidance into characterizing the growth of an individual child. A diminished response relative to prediction should lead to an overall examination of the adherence with the treatment program.

Increased adult height with treatment of GHD has been possible over the past several decades. Early diagnosis, aggressive treatment at that time, more sophisticated management of growth in the peri-pubertal period, and availability of rhGH until growth cessation have allowed children with GHD to achieve adult heights close to the mid-parental height.

In addition to an ongoing assessment of the near adult height data, there is the scrutiny of the long-term safety of GH treatment. The recognition of the devastating development of Creutzfeld-Jacob Disease in association with the use of cadaveric hGH has sensitized the current users of rhGH to potential long-term rhGH-related adverse events and the need to search for evidence of rhGH-related adverse events, such as neoplasia and cardiovascular disease.

Other indications approved for treatment with recombinant GH (rhGH) are listed below and discussed in the manuscript by Graber et al. outlining the studies that led to the approval of these indications. Children with these varied diagnoses do not have GHD, but rather have at least partial resistance to the action of hGH, perhaps without adequately increasing its production to overcome the resistance (4). To date, the FDA has approved 8 conditions in children and adolescents for which rhGH is considered both safe and effective (table):

**Table:** 

Diagnosis	Year of FDA Approval
GH deficiency	1985
Chronic renal insufficiency	1993
Turner syndrome	1996
Prader-Willi syndrome	2000
Small-for-gestational age without catch-up growth	2001
Idiopathic short stature	2003
SHOX-deficiency	2006
Noonan syndrome	2007

## Long-Acting GH

rhGH has been administered to patients as daily subcutaneous injections. This requirement leaves adherence to such regimens as an important variable when judging efficacy of treatment. Indeed, diminished adherence could falsely suggest that a given dose of rhGH was inadequate or even that a child with a given diagnosis was being treated inappropriately when growth was inadequate. The currently available LAGH preparations are in various stages of assessment (5) and it is likely that their use, when approved, will diminish the non-adherence issue.

## Growth Hormone Deficiency in Childhood Cancer Survivors

Childhood cancer survivors (CCS) are afflicted with diverse morbidities of the hypothalamic-pituitary axis due to direct effects of tumors, operative intervention, drug treatments, and radiation therapy, with radiotherapy dosage affecting the time to and intensity of the appearance of the acquired GHD. Pollock and Cohen describe the diagnosis of GHD and treatment with exogenous rhGH treatment in this population, as well as the anabolic and quality of life benefits of hGH.

Growth hormone deficiency gradually develops over post-treatment years in the setting of the ongoing risks of tumor recurrence and new secondary neoplasms. Given the mitogenic potential of rhGH and IGF-I, one must continue to assess the impact of treatment with rhGH in the survivors. There does not appear to be an increase in GH-induced tumor recurrence or secondary neoplasia, however longer term studies are needed. While the greatest tumor recurrence has been for craniopharyngioma, this is without a difference in rhGH exposure, nor an association with GH dosage (6). An increased incidence of meningiomas as secondary neoplasms in rhGH-treated patients is confounded by the strong association of meningiomas with prior brain irradiation. Thus, prior cancer therapy is not an absolute contraindication to hGH therapy. The type of primary cancer and whether the tumor occurs in the setting of a genetic syndrome associated with development of neoplasms may alter the risk-benefit ratio of rhGH therapy, although more data are needed. This is a many decades task.

## Author Contributions

All authors contributed to the drafting and editing of this editorial. All have approved the final version.

## Conflict of Interest

The authors declare that the research was conducted in the absence of any commercial or financial relationships that could be construed as a potential conflict of interest.

## Publisher’s Note

All claims expressed in this article are solely those of the authors and do not necessarily represent those of their affiliated organizations, or those of the publisher, the editors and the reviewers. Any product that may be evaluated in this article, or claim that may be made by its manufacturer, is not guaranteed or endorsed by the publisher.

## References

[1] Knobil E Morse A Wolf RC Greep RO. The Action of Bovine, Porcine and Simian Grwoth Hormone Preparations on the Costochondral Junction in the Hypophysectomized *Rhesus* Monkey. Endocrinology (1958) 62:348–54. doi: 10.1210/endo-62-3-348 13512248

[2] Raben MS. Treatment of a Pituitary Dwarf With Human Growth Hormone. J Clin Endocrinol Metab (1958) 18:901–3. doi: 10.1210/jcem-18-8-901 13563618

[3] Raiti S Tolman RA. Isolation and Purity of Human Pituitary Hormones. J Clin Endocrinol Metab (1978) 46:853. doi: 10.1210/jcem-46-5-853

[4] Storr HL Chatterjee S Metherell LA Foley C Rosenfeld RG Backeljauw PF. Non Classical Growth Hormone Insensitivity: Characteristics of Mild Abnormalities of Growth Hormone Action. Endocr Rev (2019) 40:476–505. doi: 10.1210/er.2018-00146 30265312 PMC6607971

[5] Miller BS Velazquez E Yuen KCJ. Long-Acting Growth Hormone Preparations-Current Status and Future Considerations. J Clin Endocrinol Metab (2020) 105:e2121–33. doi: 10.1210/clinem/dgz149 PMC7755139 31676901

[6] Journy NMY Zrafi WS Bolle S Fresneau B Alapetite C Allodji RS. Risk Factors of Subsequent Central Nervous System Tumors After Childhood and Adolescent Cancers: Findings From the French Cancer Center Survivor Study. Cancer Epidemiol Biomarkers Prev (2021) 30:133–41. doi: 10.1158/1055-9965.EPI-20-0735 33033142

